# COVID-19 prevalence estimation by random sampling in population - optimal sample pooling under varying assumptions about true prevalence

**DOI:** 10.1186/s12874-020-01081-0

**Published:** 2020-07-23

**Authors:** Ola Brynildsrud

**Affiliations:** 1 Norwegian Institute of Public Health, Oslo, Norway; 2 Norwegian University of Life Science, Ås, Norway

## Abstract

**Background:**

The number of confirmed COVID-19 cases divided by population size is used as a coarse measurement for the burden of disease in a population. However, this fraction depends heavily on the sampling intensity and the various test criteria used in different jurisdictions, and many sources indicate that a large fraction of cases tend to go undetected.

**Methods:**

Estimates of the true prevalence of COVID-19 in a population can be made by random sampling and pooling of RT-PCR tests. Here I use simulations to explore how experiment sample size and degrees of sample pooling impact precision of prevalence estimates and potential for minimizing the total number of tests required to get individual-level diagnostic results.

**Results:**

Sample pooling can greatly reduce the total number of tests required for prevalence estimation. In low-prevalence populations, it is theoretically possible to pool hundreds of samples with only marginal loss of precision. Even when the true prevalence is as high as 10% it can be appropriate to pool up to 15 samples. Sample pooling can be particularly beneficial when the test has imperfect specificity by providing more accurate estimates of the prevalence than an equal number of individual-level tests.

**Conclusion:**

Sample pooling should be considered in COVID-19 prevalence estimation efforts.

## Background

It is widely accepted that a large fraction of COVID-19 cases go undetected. A crude measure of population prevalence is the fraction of positive tests at any given date. However, this is subject to large ascertainment bias since tests are typically only ordered from symptomatic cases, whereas a large proportion of infected might show little to no symptoms [1, 2]. Non-symptomatic infections can still shed the Severe acute respiratory syndrome coronavirus 2 (SARS-CoV-2) virus and are therefore detectable by reverse transcriptase polymerase chain reaction (RT-PCR)-based tests. It is therefore possible to test randomly selected individuals to estimate the true disease prevalence in a population. However, if the disease prevalence is low, very little information is garnered from each individual test. Under such situations it can be advantageous to pool individual patient samples into a single pool [3–5]. Pooling strategies, also called group testing, effectively increase the test capacity and reduces the required number of RT-PCR-based tests. For SARS-CoV-2 pooling has been estimated to potentially reduce costs by 69% [6], use ten-fold fewer tests [7] and clearing 20 times the number of people from isolation with the same number of tests [8]. Note that I will not discuss pooling of SARS-CoV-2 antibody-based tests, since there is currently not enough information about how pooling affects test parameters. However, sample pooling has been successfully used for seroprevalence studies for other diseases such as human immunodeficiency virus (HIV) [9–11].

## Methods

I simulated the effect sample pooling had on prevalence estimates under five different settings for true prevalence, *p*. I started by generating a population of 500,000 individuals and then let each individual have *p* probability of being infected at sampling time. The number of patient samples collected from the population is denoted by *n*, and the number of patient samples that are pooled into a single well is denoted by *k*. The total number of pools are thus $\frac{n}{k}$, hereby called *m*. The number of positive pools in an experiment is termed *x*. I calculated the estimated prevalence $\hat{p}$ at each parameter combination by replicating the experiment 100,000 times and report here the 2.5 and 97.5% quantiles of the distribution of $\hat{p}$.

Explored parameter options:

$$ p\in \left\{0.001,0.003,0.01,\mathrm{0.03,1.0}\right\} $$

$$ n\in \left\{200,500,1000,1500,2000,3000,5000\right\} $$

$$ k\in \left\{1,3,5,7,10,15,20,25,30,40,50,70,100,200\right\} $$

I considered the specificity (*θ*) of a PCR-based test to be 1.0 but include simulations with the value set to 0.99. Test sensitivity (*η*) depends on a range of uncontrollable factors such as virus quantity, sample type, time from sampling, laboratory standard and the skill of personnel [12]. There have also been reports of it varying with pooling level [13]. For the purposes of this study, I fixed the sensitivity first at 0.95, then at 0.7, irrespective of the level of pooling. These estimates are rather low, which would suggest that I am somewhat overestimating the uncertainty of $\hat{p}$. However, since it is possible that tests will be carried out under suboptimal and non-standardized conditions I prefer to err on the side of caution.

A central point of pooled testing is that the number of positive pools, *x*, divided by the total number of pools, *m*, can be used as a proxy to measure the true prevalence when the test sensitivity and specificity is known. Note that the number of positive pools, *x*, can be approximated in infinite populations as a stochastic variable subject to a binomial distribution with parameters *m* and *P*, where the latter is the probability that a single pool will test positive. A positive pool can arise from two different processes: There can be one or more true positive samples in the pool, and they are detected, or there can be no true positive samples in the pool, but the test gives a false positive result. These two possibilities are represented by the first and second part of the following equation [14], respectively:

1 $$ P\left(p,k\right)=\left(1-{\left(1-p\right)}^k\right)\eta +{\left(1-p\right)}^k\left(1-\theta \right) $$

Closer inspection of the above formula reveals something disheartening: When *p* approaches zero, *P* converges towards 1 − *θ*. Thus, in low-prevalence scenarios, and for typical values of test sensitivity and specificity, most positive test results will be false positives. Nevertheless, with appropriate levels of sample pooling it is possible to get decent estimates of the true prevalence because the probability of having no positive samples in a pool decreases with *k*.

We can modify eq. 1 for finite populations by replacing *P* with $\frac{x}{m}$, *p* with $\hat{p}$, and then solving for $\hat{p}$. This gives us the formula of Cowling et al., 1999 [15], which is used in the following to calculate $\hat{p}$ from a single sample:

2 $$ \hat{p}=1-{\left(\frac{\eta -\frac{x}{m}}{\theta +\eta -1}\right)}^{\frac{1}{k}} $$

Note that the formula incorporates the test parameters and thus gives an unbiased estimate of $\hat{p}$ even in low-prevalence settings. In this formula, x is a stochastic variable with a binomial distribution. It depends on the number of truly positive samples in a pool, another stochastic variable with a binomial distribution. As a final layer of complexity, we can take samples from a finite population. For these reasons I will use Monte Carlo simulations to get estimates for $\hat{p}$ rather than evaluating some closed-form mathematical expression.

### An algorithm for patient-level diagnosis

**Fig. 1:**
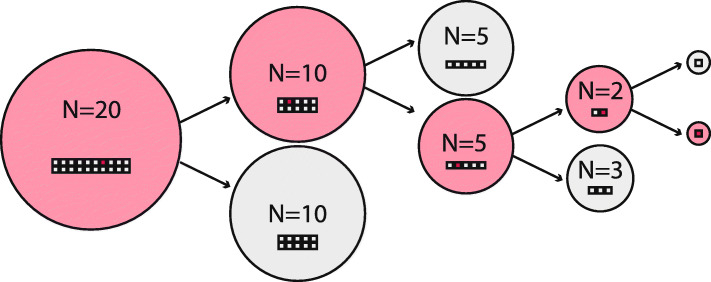
Algorithm used to minimize the number of RT-PCR reactions in pooled sampling. Negative pools regard all constituent patient samples as negative, whereas positive pools are split in two, and the process repeated. Red circle = Pool testing positive. Grey circle = Pool testing negative. Red/grey squares = Patient samples in pool, with color indicating diseased/non-diseased status

A crucial objective of testing is to identify which patients have active COVID-19 infections. This information is not readily apparent from pooled tests, and in order to get diagnostic results at the patient level, some samples will need to be retested. The methodologically simplest algorithm is to consider all samples from negative pools as true negatives, but re-test every sample from a positive pool individually. This is also called Dorfman’s method [4]. This strategy is estimated to increase testing capabilities by at least 69% [6]. In this work I use an algorithm that conserves testing resources even more than this, but which might be more difficult to implement in practice: I remove all samples from negative pools, considering them true negatives. All positive pools are split into two equally large sub-pools, and then the process is repeated. Positive patient-level diagnosis is only made from sub-pools of size 1. The algorithm is illustrated in Fig. 1. Note that this is a sub-optimal version of the generalized binary splitting (GBS) algorithm presented in the context of COVID-19 in [16]. My version is sub-optimal in the number of reactions because I am always running a test on both sub-pools when a parent pool has tested positive. It is possible to run an even lower number of reactions by not testing a sub-pool if the other sub-pool from the same parent pool has been run first and tested negative. (The positive result from the parent pool implies that the second sub-pool must be positive.) However, for practical reasons such as the ability to run multiple tests simultaneously and the fact the tests are imperfect, I have used the algorithm in Fig. 1. A thorough discussion on group testing algorithms and their merit in testing for SARS-CoV-2 is available in [7].

## Results

### Estimates of prevalence

**Fig. 2:**
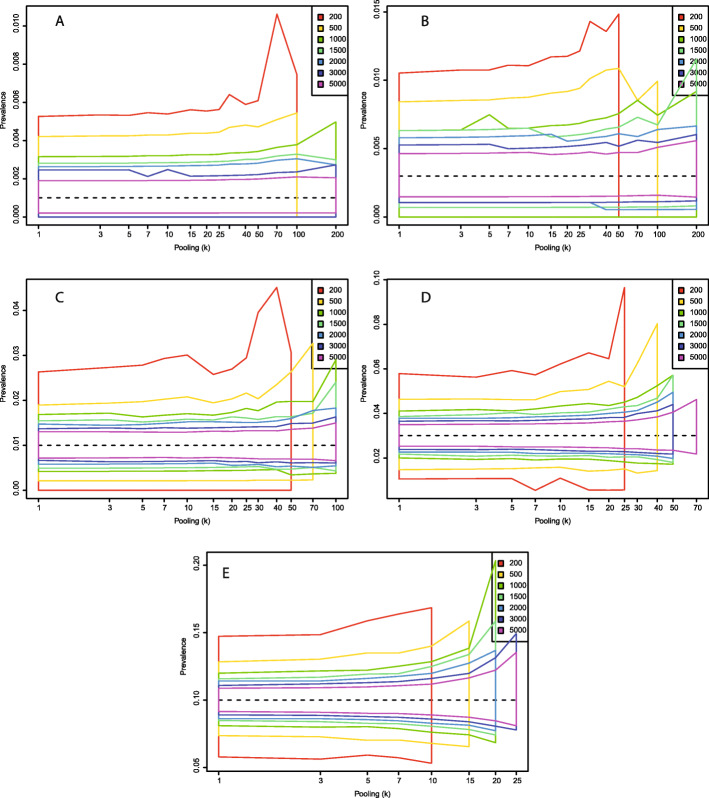
Central 95% estimates of $\hat{p}$ with a test with sensitivity (*η*) 0.95 and perfect specificity (*θ* = 1) under different combinations of total number of samples and level of sample pooling. **a**: *p* = 0.001; **b**: *p* = 0.003; **c**: *p* = 0.01; **d**: *p* = 0.03; **e**: *p* = 0.10

**Fig. 3:**
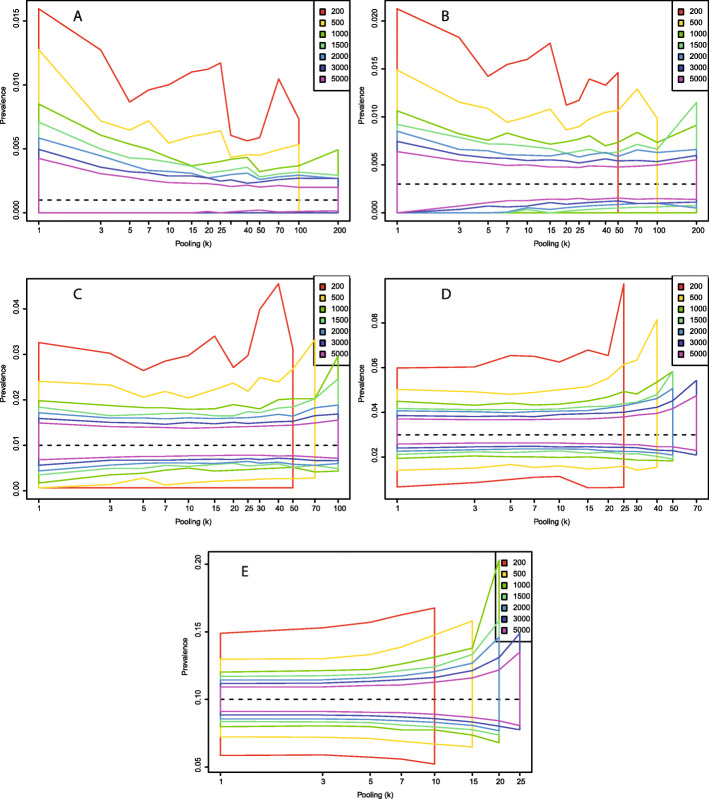
Central 95% estimates of $\hat{p}$ with a test with sensitivity (*η*) 0.95 and a specificity (*θ*) of 0.99 under different combinations of total number of samples and level of sample pooling. **a**: *p* = 0.001; **b**: *p* = 0.003; **c**: *p* = 0.01; **d**: *p* = 0.03; **e**: *p* = 0.10

**Fig. 4:**
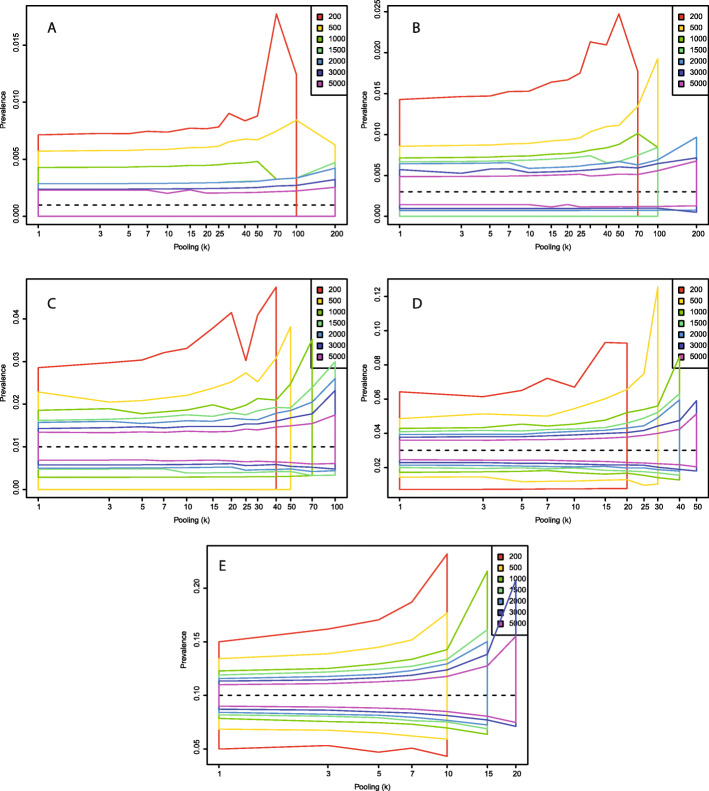
Central 95% estimates of $\hat{p}$ with a test with sensitivity (*η*) 0.70 and perfect specificity (*θ* = 1) under different combinations of total number of samples and level of sample pooling. **a**: *p* = 0.001; **b**: *p* = 0.003; **c**: *p* = 0.01; **d**: *p* = 0.03; **e**: *p* = 0.10

**Fig. 5:**
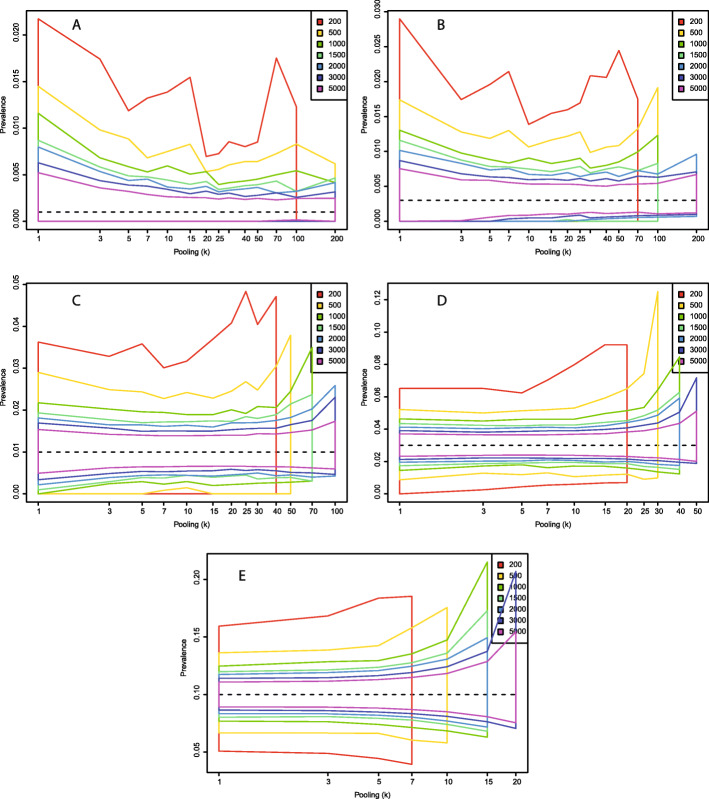
Central 95% estimates of $\hat{p}$ with a test with sensitivity (*η*) 0.70 and a specificity (*θ*) of 0.99 under different combinations of total number of samples and level of sample pooling. **a**: *p* = 0.001; **b**: *p* = 0.003; **c**: *p* = 0.01; **d**: *p* = 0.03; **e**: *p* = 0.10

In the following, I use simulations to calculate the central 95% estimates of $\hat{p}$ using tests with varying sensitivity (0.7 and 0.95) and specificity (0.99 and 1.0) (Figs. 2, 3, 4, 5). These estimates are based on the initial pooled tests only, not the follow-up tests on sub-pools that allow for patient-level diagnosis. (Including results from these samples would allow the precision from the pooled test estimates to approach those of testing individually.) More samples are associated with a distribution of $\hat{p}$ more narrowly centered around the true value, while higher levels of pooling are generally associated with higher variance in the $\hat{p}$ estimates. The latter effect is less pronounced in populations with low prevalence. For example, if the true population prevalence is 0.001 and a total of 500 samples are taken from the population, the expected distribution of $\hat{p}$ is nearly identical whether samples are run individually (*k* = 1) or whether they are run in pools of 25 (Figs. 2 or 4, panel A). Thus, it is possible to economize lab efforts by reducing the required number of pools to be run from 500 to 20 (500 divided by 25) without any significant alteration to the expected distribution of $\hat{p}$. At this prevalence and with this pooling level, 40 tests are sufficient to get a correct patient-level diagnosis for all 500 individuals 97.5% of the time (Supplementary Table 1). With 5000 total samples, the central estimates of $\hat{p}$ vary little between individual samples (95% interval 0.00021–0.0021) and a pooling level of 200 (95% interval 0.0022–0.0021). 145 reactions is enough to get patient-level diagnosis 97.5% of the time, in other words a reduction in the number of separate RT-PCR setups by a factor of 34.5. (Supplementary Table 1).

The situation changes when the test specificity (*θ*) is set to 0.99, that is, allowing for false positive test results (Figs. 3, 5). This could theoretically occur from PCR cross-reactivity between COVID-19 and other viruses, or from human errors in the lab. A problem with imperfect specificity tests are that false positives typically outnumber true positives when the true prevalence is low. This creates a seemingly paradoxical situation in which higher levels of sample pooling often leads to prevalence estimates that are more accurate. This is because many pools test positive without containing a single true positive sample, leading to inflated estimates of the prevalence. When the level of pooling goes up, the probability that a positive pool contains at least one true positive sample increases, which increases the total precision. The trends about appropriate levels of pooling for different sample numbers and levels of true population prevalence are similar as for the perfect specificity scenario, but with imperfect specificity, we have an added incentive for sample pooling in that prevalence estimates are closer to the true value with higher levels of pooling. Even with a moderately accurate test (sensitivity 0.7 and specificity 0.99), when the prevalence is 1%, pooling 50 together lets us diagnose 5000 individuals at the patient-level with a median of 282 tests, a 17-fold reduction in the number of tests. This has virtually no influence on our estimate of $\hat{p}$, and no significant effect on the number of wrongly diagnosed patients, which in both cases is about 1%.

## Discussion

The relationship between true prevalence, total sample number and level of pooling is not always intuitive. Some combinations of parameters have serrated patterns for $\hat{p}$, which looks like Monte Carlo errors (Figs. 2, 3, 4, and 5). This is particularly true for the lower sample counts. However, this is not due to stochasticity, but due to the discrete nature of each estimate of $\hat{p}$. That is, $\hat{p}$ is not continuous and for small pool sizes miniscule changes in the number of positive pools can affect the estimate quite a bit.

For example, if we take 200 samples and go with a pool size of 100, there are only three potential outcomes: First, both pools are negative, in which case we believe the prevalence is 0. Second, one pool is positive and the other negative, in which case we estimate $\hat{p}$ as approximately 0.007 if the test sensitivity is 0.95. Finally, both pools are positive, in which case the formula of Cowling et al. does not provide an answer because the fraction of positive pools is higher than the test sensitivity. This formula is only intended to be used when the fraction of positive pools is much lower than the test sensitivity.

In general, very high levels of pooling are not appropriate since, depending on the true prevalence, the probability that every single pool has at least one positive sample approaches 1. (Indicated by “NA” in Supplementary Table 1). In low prevalence settings however, it can be appropriate to pool hundreds of samples, but the total number of samples required to get a precise estimate of the prevalence is much higher. Thus, decisions about the level of pooling need to be informed by the prior assumptions about prevalence in the population, and there is a prevalence-dependent sweet spot to be found in the tradeoff between precision and workload.

It is worth noting that the strategy I have outlined here does present some logistical challenges. Firstly, samples must be allocated to pools in a random manner. This rules out some practical approaches such as sampling a particular sub-district and pooling these, then sampling another district the next day. Secondly, binary testing of sub-pools might be more cumbersome than it’s worth, in which case Dorfman’s method should be preferred. Finally, there are major organizational challenges related to planning and conducting such experiments across different testing sites and jurisdictions.

## Conclusion

Attempts to estimate the true current prevalence of COVID-19 by PCR tests can benefit from sample pooling strategies. Such strategies have the potential to greatly reduce the required number of tests with only slight decreases in the precision of prevalence estimates. If the prevalence is low, it is generally appropriate to pool even hundreds of samples, but the total sample count needs to be high in order to get reasonably precise estimates of the true prevalence. On the other hand, if the prevalence is high there is little to be gained by pooling more than 15 samples. Pooling strategies makes it possible to get patient-level diagnostic information with only a fraction of the number of tests as individual testing. For a prevalence of 10%, pooling cut the required number of tests by about two thirds, while for a prevalence of 0.1%, the number of required tests could on average be lowered by a factor of 50.

## Supplementary information

**Additional file 1 : Supplementary Table 1**. Table containing prevalence estimates and, the estimated required number of tests, and the expected proportion incorrectly classified patients for all parameter combinations. Se = sensitivity. Sp = specificity. N = number of samples. k = pooling level. P = true prevalence. *p* 2.5%, *p* 50.0%, *p* 97.5% = 2.5, 50 and 97.5 quantile of estimated prevalence. *T* 2.5%, *T* 50.0%, *T* 97.5% = 2.5, 50 and 97.5 quantile of estimated number of tests required to get individual-level diagnoses. E(S) = Expected number of tests saved when compared to testing individually for this N. E(inc) = Expected percentage of patients that are diagnosed incorrectly at this parameter combination. [Excel file].

**Additional file 2 : Supplementary document 1**. Testing for freedom from disease and distinguishing a disease-free population from a low-prevalence one.

**Additional file 3 : Figure S1**. Testing for freedom of disease with a test with perfect specificity. The x-axis represents different true levels of *p*, and the colored lines represent the number of samples associated with 95% probability of having at least one positive sample at that prevalence level. For perfect specificity tests this is commonly interpreted as meaning that we can be 95% certain that the true prevalence is lower. The effects of sample pooling are explored with different color lines. Panel A: Test specificity = 1.0; Panel B: Test specificity = 0.99.

**Additional file 4 : Figure S2**. Using a test with specificity of 0.99 to discriminate a disease-free population from a population with *p* = 0.005 with 2743 samples from both populations. Panel A: The expected number of positive samples from the disease-free and the low-prevalence populations; Panel B: The probability mass function of the difference in the number of positive samples between the low-prevalence and the disease-free population. With 2743 samples from both populations, there is a 5% probability of getting more positive tests from the disease-free population.

## Data Availability

Code written for this project is available at https://github.com/admiralenola/pooledsampling-covid-simulation. All simulations and plots were created in R version 3.2.3 [17].

## Abbreviations

HIV: Human immunodeficiency virus
RT-PCR: Reverse transcriptase polymerase chain reaction
SARS-CoV-2: Severe acute respiratory syndrome coronavirus 2
