# Supplementary material for: COVID-19 prevalence estimation by random sampling in population - optimal sample pooling under varying assumptions about true prevalence
Source: BMC Med Res Methodol. 2020 Jul 23;20:196. doi: 10.1186/s12874-020-01081-0 (PMC7376319; doi:10.1186/s12874-020-01081-0)
Supplement: Supplementary file 2 — Additional file 2 : Supplementary document 1. Testing for freedom from disease and distinguishing a disease-free population from a low-prevalence one. [file 12874_2020_1081_MOESM2_ESM.docx]

**COVID-19 prevalence estimation by random sampling in population - Optimal sample pooling under varying assumptions about true prevalence – SUPPLEMENTARY MATERIAL**

*Ola Brynildsrud ^1,2^*

^1^ Norwegian Institute of Public Health, Oslo, Norway

^2^ Norwegian University of Life Science, Ås, Norway

Contact: olbb@fhi.no

**Freedom from disease and low-prevalence populations**

Pooled sampling can also be used to efficiently assert freedom from disease with a certain probability. If the population is free from the disease, then we find no truly positive sample. The question then becomes how many samples we need to take from a population with prevalence $p$ to ensure that the probability of sampling at least one single positive patient is $\alpha$ or higher. I calculated this number using the formula of Christensen and Gardner, 2000 [[1]](https://paperpile.com/c/jepAok/7Wv3):

|  | $n\geq\frac{log(1-(1-\alpha))}{log(\theta(1-p) + (1-\eta)p)}$ | (3) |
| --- | --- | --- |

The formula of Christensen and Gardner can be modified to accommodate pooled sampling:

|  | $m\geq\frac{log(1-(1-\alpha))}{log({(1-p)}^{k}\theta+ (1-{(1-p)}^{k})(1-\eta)}$ | (4) |
| --- | --- | --- |

When the prevalence is zero, the formula becomes much simpler:

|  | $m\geq\frac{log(\alpha))}{\log\left( \theta\right)} ,\theta\neq1$ | (5) |
| --- | --- | --- |

True freedom in a population is not possible to assert without sampling every individual. However, we can establish how many samples we need in order to have at least $(1-\alpha)\%$ probability of getting a positive sample if the true prevalence was $p$. For example, from Fig. S1, panel A, we can see that by sampling 60 patients from a population with a true prevalence of 0.06, we would be 95% certain that at least one of our samples came out positive. That is, if the true prevalence in the source population was exactly 0.06, we would only expect to get 60 negative samples by chance 5% of the time. A common interpretation of this is that if all pools test negative, we can be 95% certain that the true prevalence in the source population is 0.06 or lower.

Note that when the specificity is 1.0, we will never get a positive test from a completely disease-free population no matter how many samples we take. However, if the test specificity is less than 1.0, the sample size needed to ensure $(1-\alpha)\%$ probability of getting at least one positive sample has an upper bound, even when the population is free of disease. This is the sample size for which we would expect at least a $\alpha\%$ probability of getting a false positive result (Fig. S1, panel B). From equation (3) this number is 299 for an $\alpha$ of 5% and a specificity of 0.99.

When tests have imperfect specificity $(\theta\neq1)$, it requires a high number of samples to distinguish between a truly disease-free population and one with low prevalence. One way to calculate how many samples are needed to distinguish between the two is to see it as a classical statistical hypothesis testing problem. If we let $X$ be the number of positive samples from a disease-free population, and $Y$ the number of positive samples from a population with low prevalence, we can analyze the probability mass function of the difference between these two binomial variables to see what sample numbers are required to reject the null hypothesis that $X=Y$.

|  | $X \sim BIN\left( n,P_{1} \right), P_{1}=(1-\theta)$ | (6) |
| --- | --- | --- |
|  | $Y \sim BIN\left( n,P_{2} \right), P_{2}=p\mu+(1-\theta)(1-p)$ | (7) |
|  | $Z=X-Y$ | (8) |

Mathematically, the probability mass function of $Z$ can be calculated as follows:

|  | $p\left( z \right)= \left\{ \begin{aligned} \sum_{i=0}^{n} \binom{n}{i+z}{P_{1}}^{i+z}{(1-P_{1})}^{n-(i+z)}\binom{n}{i}{P_{2}}^{i}{(1-P_{2})}^{n-i}, if z\geq0 \\ \sum_{i=0}^{n} \binom{n}{i}{P_{1}}^{i}{(1-P_{1})}^{n-i}\binom{n}{i+z}{P_{2}}^{i+z}{(1-P_{2})}^{n-(i+z)}, otherwise \end{aligned} \right.$ | (9) |
| --- | --- | --- |

Using formula (9), we find that with a test specificity of 0.99, taking 2743 samples from a disease-free population and as many from a population with a prevalence of 0.005, we will have a 5% probability of getting *more* positive samples from the disease-free population than from the low-prevalence one (Fig. S2). If the true prevalence drops to 0.001, the required number of samples to reliable differentiate from a disease-freeness 95% of the time jumps to nearly 70000. With this is mind, it is hard to imagine a situation in which random testing with an imperfect specificity test could be used to demonstrate true freedom rather than a very low prevalence.

**REFERENCES**

1. Christensen J, Gardner IA. Herd-level interpretation of test results for epidemiologic studies of animal diseases. Preventive Veterinary Medicine. 2000. pp. 83–106. doi:10.1016/s0167-5877(00)00118-5

**FIGURE LEGENDS**

Fig. S1 – Testing for freedom of disease with a test with perfect specificity. The x-axis represents different true levels of $p$, and the colored lines represent the number of samples associated with 95% probability of having at least one positive sample at that prevalence level. For perfect specificity tests this is commonly interpreted as meaning that we can be 95% certain that the true prevalence is lower. The effects of sample pooling are explored with different color lines. Panel A: Test specificity = 1.0; Panel B: Test specificity = 0.99.

Fig. S2 – Using a test with specificity of 0.99 to discriminate a disease-free population from a population with $p=0.005$ with 2743 samples from both populations. Panel A: The expected number of positive samples from the disease-free and the low-prevalence populations; Panel B: The probability mass function of the difference in the number of positive samples between the low-prevalence and the disease-free population. With 2743 samples from both populations, there is a 5% probability of getting more positive tests from the disease-free population.
