# Supplementary figures and images for: COVID-19 prevalence estimation by random sampling in population - optimal sample pooling under varying assumptions about true prevalence
Source: BMC Med Res Methodol. 2020 Jul 23;20:196. doi: 10.1186/s12874-020-01081-0 (PMC7376319; doi:10.1186/s12874-020-01081-0)

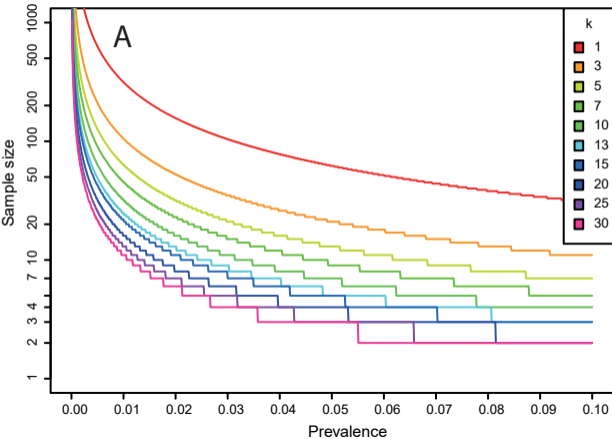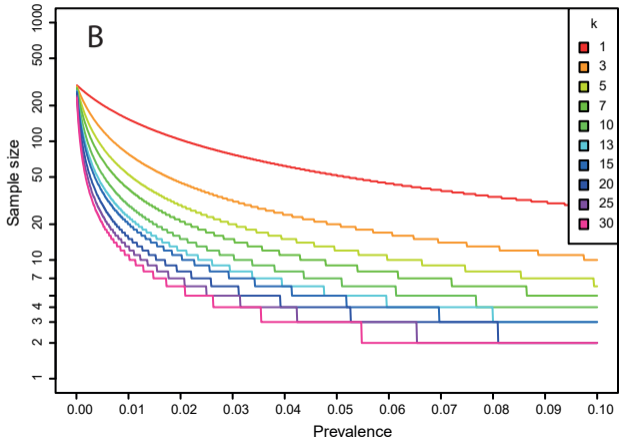

Supplement: Supplementary file 3 — Additional file 3 : Figure S1. Testing for freedom of disease with a test with perfect specificity. The x-axis represents different true levels of p, and the colored lines represent the number of samples associated with 95% probability of having at least one positive sample at that prevalence level. For perfect specificity tests this is commonly interpreted as meaning that we can be 95% certain that the true prevalence is lower. The effects of sample pooling are explored with different color lines. Panel A: Test specificity = 1.0; Panel B: Test specificity = 0.99. [file 12874_2020_1081_MOESM3_ESM.pdf]

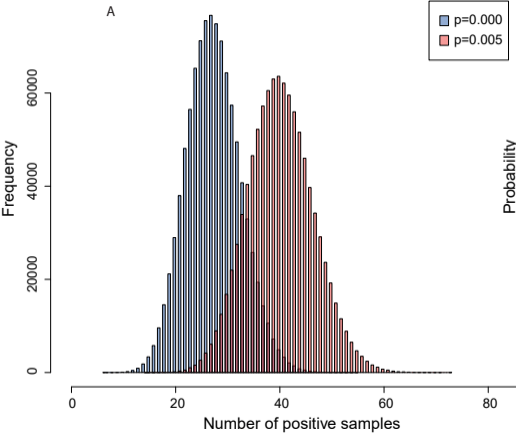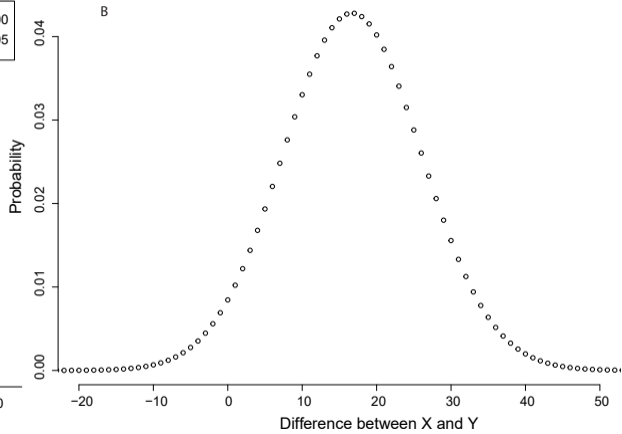

Supplement: Supplementary file 4 — Additional file 4 : Figure S2. Using a test with specificity of 0.99 to discriminate a disease-free population from a population with p = 0.005 with 2743 samples from both populations. Panel A: The expected number of positive samples from the disease-free and the low-prevalence populations; Panel B: The probability mass function of the difference in the number of positive samples between the low-prevalence and the disease-free population. With 2743 samples from both populations, there is a 5% probability of getting more positive tests from the disease-free population. [file 12874_2020_1081_MOESM4_ESM.pdf]
